# METTL3 promotes esophageal squamous cell carcinoma progression and reduces chemosensitivity to paclitaxel through the CASP9/BIRC3-dependent apoptosis pathway

**DOI:** 10.1016/j.gendis.2025.101693

**Published:** 2025-05-27

**Authors:** Pengxiang Ji, Bo Wan, Minghui Gao, Shaohua Yin, Han Wu, Junjie Wang, Yuting Ma, Weihua Xu, Minghua Wang

**Affiliations:** a Department of Biochemistry and Molecular Biology, Suzhou Medical College of Soochow University, Suzhou, Jiangsu 215123, China; b School of Life Sciences, Suzhou Medical College of Soochow University, Suzhou, Jiangsu 215123, China; c Suzhou Ruihua Orthopedic Hospital, Suzhou, Jiangsu 215104, China; d CAS Key Lab of Bio-Medical Diagnostics, Suzhou Institute of Biomedical Engineering and Technology, Chinese Academy of Sciences, Suzhou, Jiangsu 215163, China; e Department of Thoracic and Cardiac Surgery, The Second Affiliated Hospital of Soochow University, Suzhou, Jiangsu 215004, China; f State Key Laboratory of Chemical Oncogenomics, Key Laboratory of Chemical Genomics, Peking University Shenzhen Graduate School, Shenzhen, Guangdong 518055, China

Epigenetic alteration is one of the common features in cancer progression.^1^ N6-methyladenosine (m^6^A) RNA modification regulates RNA metabolism and has been implicated in the development and progression of cancers.^2^ In our study, we discovered that the expression of methyltransferase-like 3 (METTL3) was significantly elevated in human esophageal squamous cell carcinoma (ESCC) tissues. Ablation of METTL3 inhibited proliferation and migration and induced apoptosis in ESCC cells both *in vitro* and *in vivo*. Paclitaxel (PTX) treatment resulted in a significant up-regulation of METTL3 expression within ESCC cells. Mechanistically, METTL3 promoted ESCC development and reduced chemosensitivity to PTX through regulating the mRNA stability of apoptosis-related genes caspase 9 (CASP9) and apoptosis protein repeat-containing 3 (BIRC3). These findings reveal the molecular mechanism of METTL3 in ESCC development and progression, providing new insights for developing molecular diagnosis and therapies for this malignancy.

In our investigation of RNA m^6^A methylation regulators in ESCC, we found that METTL3 was significantly up-regulated in ESCC tissues compared with normal tissues among the Cancer Genome Atlas Program (TCGA) data and confirmed by our data (*n* = 21; *p* = 0.00275; Fig. S1). These results indicate that the dysregulation of RNA m^6^A methylation, particularly the elevated expression of METTL3, may play a role in ESCC pathogenesis.

To explore the biological effects of METTL3 on ESCC cells, we utilized CRISPR/Cas9 technology to generate METTL3-knockout ESCC cell lines, Eca-109 and CaEs-17 (Table S1 and Fig. S2A, B). Our experimental data from CCK-8, crystal violet, and transwell assays indicated a substantial decrease in the proliferation and migration of METTL3-knockout cells relative to controls (*n* = 4; *p* < 0.05; Fig. S2C–F). In parallel, the METTL3-knockout cells exhibited a significant reduction in m^6^A methylation levels in their total RNA (Fig. S2B). Notably, the re-expression of METTL3 effectively rescued the proliferation and migration defects induced by METTL3 deficiency (Fig. 1A–D). Furthermore, flow cytometry analysis showed that the apoptosis levels were significantly increased in Eca-109 and CaES-17 cell lines with METTL3 knockout compared with the control cells (*n* = 3; *p* < 0.05; Fig. 1E; Fig. S3C, D).

**Figure 1:**
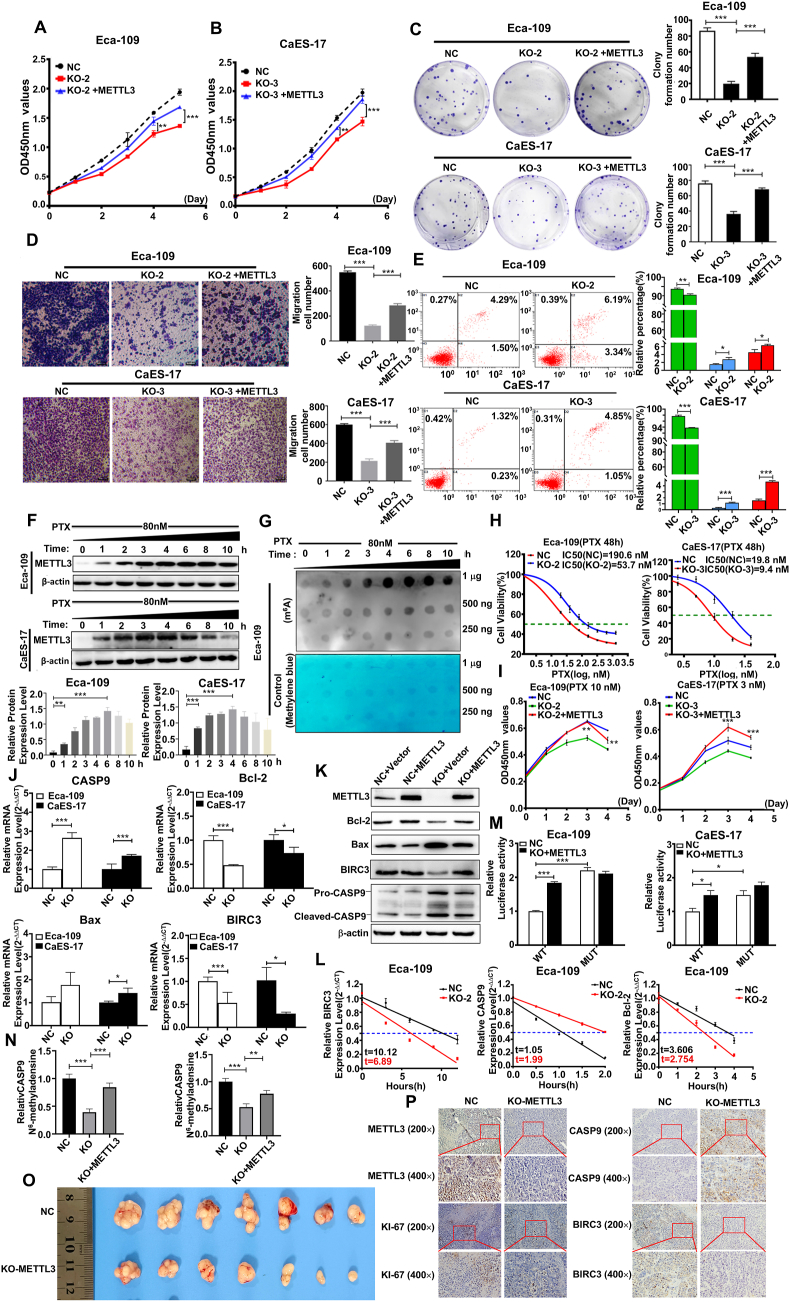
METTL3 promotes esophageal squamous cell carcinoma progression and reduces chemosensitivity to paclitaxel (PTX) through the CASP9/BIRC3-dependent apoptosis pathway. **(A, B)** CCK-8 was used to detect the cell proliferation ability in the NC (Eca-109-WT cells) group, the KO (METTL3-KO cells) group, and the KO + METTL3 (METTL3-KO cells restored with METTL3) group in Eca-109 and CaES-17 cells (*n* = 4). **(C)** Crystal violet was used to detect the number of cell colonies formed in the NC group, the KO group, and the KO + METTL3 group in Eca-109 and CaES-17 cells (*n* = 3). **(D)** Transwell assay was used to detect the cell migration levels of the NC group, the KO group, and the KO + METTL3 group in Eca-109 and CaES-17 cells (*n* = 3). **(E)** Annexin V-FITC/PI apoptosis flow cytometry analysis was used to detect the apoptosis level in wild-type and METTL3-KO ESCC cells (*n* = 3). **(F)** Western blotting was used to detect the expression level of METTL3 protein in Eca-109 and CaES-17 cells after exposure to PTX (80 nM, *n* = 3). **(G)** Dot blotting was used to detect the m^6^A methylation level in the Eca-109 cell line after PTX treatment. **(H)** IC50 values of PTX treatment on wild-type (NC) and METTL3-KO ESCC cell lines (*n* = 4). **(I)** Proliferation curves of wild-type (NC), METTL3-KO, and KO + METTL3 ESCC cells exposed to PTX (*n* = 3). **(J)** Quantitative PCR was used to detect the mRNA levels of apoptosis-related genes in wild-type and METTL3-KO ESCC cells (*n* = 3). **(K)** Western blotting analysis of apoptosis-related gene expression in wild-type (NC), METTL3-KO, and KO + METTL3 ESCC cells. **(L)** The T_1/2_ of the target genes' mRNA (CASP9, Bcl-2, BIRC3) were detected after actinomycin D treatment in wild-type and METTL3-KO Eca-109 cells (*n* = 3). **(M)** CASP9 3′-UTR luciferase activity in Eca-109 and CaES-17 cell lines (*n* = 3). **(N)** CASP9 m^6^A modification levels in wild-type and METTL3-KO ESCC cells were determined using methylated RNA immunoprecipitation/m^6^A-quantitative PCR (*n* = 3). **(O)** METTL3 knockdown inhibited the growth of subcutaneous xenografts *in vivo* (*n* = 7). **(P)** Immunohistochemical staining results (METTL3, Ki-67, CASP9, and BIRC-3) in xenograft tissues. The data were presented as mean ± standard deviation. ∗*p* < 0.05, ∗∗*p* < 0.01, and ∗∗∗*p* < 0.001.

PTX is an active therapeutic agent against ESCC, while the PTX insensitivity and resistance occur frequently in treating ESCC; the mechanism is not fully understood yet.^3^ We found that the level of METTL3 expression and m^6^A-RNA methylation in ESCC cells was induced by PTX in a time-dependent manner (Fig. 1F). Notably, ablation of METTL3 in ESCC cells exhibited increased sensitivity to PTX, accompanied by an elevated rate of apoptosis (Fig. S3E). The half-maximal inhibitory concentration (IC50) of PTX in the Eca-109-WT (NC) cell was 109.60 nM, which was 3.5 times higher than that in the EMTTL3-knockout Eca-109 cells (53.72 nM). The IC50 of PTX was also significantly lower in METTL3-knockout CaEs-17 cells (9.40 nM) compared with CaEs-17-WT cells (19.83 nM) (Table S2, Fig. 1H). When METTL3 was re-expressed in METTL3-knockout ESCC cells, the sensitivity to PTX was comparable to that in wild-type Eca-109 and CaEs-17 cells (Fig. 1I). These data suggest a link between METTL3 expression and insensitivity to PTX in ESCC cells.

In our quantitative analysis of apoptosis-related gene expression in ESCC cells, we found that there were relatively significant changes in four genes between the METTL3-knockout and NC groups, among which Bcl-2 and BIRC3 were down-regulated, while Bax and CASP9 were up-regulated in METTL3-knockout cells (Table S3 and Fig. S4A; Fig. 1J). In the Eca-109 cells treated with actinomycin D (15 μg/mL), the T_1/2_ (half-life) of CASP9 mRNA was significantly shortened in METTL3-knockout cells compared with the control cells, while the T_1/2_ of Bcl-2 and BIRC3 mRNA were raised in METTL3-knockout cells (Fig. 1L).

To determine whether CASP9 was a direct target of METTL3. Analysis of the m^6^Avar database (http://m6avar.renlab.org/) revealed one m^6^A motif located in the 3′ untranslated region (3′UTR) of CASP9 (#1) for m^6^A modification (Fig. S4B, C). We constructed CASP9 3′UTR luciferase reporters containing wild-type m^6^A site or mutant-type (A-C substitution), to elucidate the specific influence of m^6^A methylation on CASP9 gene expression (Fig. S4B, C). The luciferase activity of the mutant-type was significantly higher compared with the wild-type in Eca-109 and CaES-17 cells. Additionally, the luciferase activity of the mutant-type reporter was observed to be markedly elevated in METTL3-knockout cells relative to the control cells (Fig. 1M).

The result of methylated RNA immunoprecipitation/m^6^A-quantitative reverse transcription PCR showed that anti-m^6^A antibody significantly enriched CASP9 and BIRC3 mRNA levels in Eca-109 cells (*n* = 3; *p* < 0.05; Fig. 1N). Western blotting analysis revealed that the METTL3 ablation led to a decrease in the expression of Bcl-2 and BIRC3, while simultaneously increasing Bax and CASP9 expression (Fig. 1K).

METTL3 overexpression enhanced BIRC3 and Bcl-2 expression or decreased CASP9 expression in a dose-dependent fashion in Eca-109 cells (Fig. S4D). Moreover, PTX treatment could dramatically increase the expression of METTL3 or decrease the expression of CASP9 in Eca-109 cells in a time-dependent manner, while these significant changes of CASP9 and BIRC3 expression were not observed in METTL3-knockout Eca-109 cells (Fig. S4E, F). These data indicate that the expression of CASP9 and BIRC3 was under the control of METTL3-associated m^6^A modification.

To investigate whether the METTL3 gene worked as an oncogene in ESCC *in vivo*, we established the xenograft model using nude mice. Our result revealed that the ablation of METTL3 significantly inhibited tumor formation and reduced the tumor weight (*n* = 7; *p* < 0.05), consistent with the results demonstrating that METTL3 knockout inhibited the cell proliferation of ESCC cells *in vitro* (Fig. 1O; Fig. S5A–C). To further elucidate the role of the METTL3-apoptosis pathway in driving the progression of ESCC, we also detected the expression of CASP9 and BIRC3 in the xenograft model with METTL3-ablated ESCC and control cells. The results indicated that METTL3, BIRC3, and MKI67 were down-regulated, while CASP9 was up-regulated in tumors derived from METTL3-knockout ESCC cells injected into nude mice (Fig. 1P).

In summary, our study compellingly illustrates that the overexpressed METTL3 in ESCC, the major methyltransferase catalyzing m^6^A modification of mRNA, disrupts the delicate balance of m^6^A modification. This disruption is considered a critical factor contributing to the proliferation, metastasis, and PTX insensitivity of ESCC cells. CASP9 is a key player in the intrinsic or mitochondrial pathway of apoptosis, which is involved in various stimuli, including chemotherapies, stress agents, and radiation.^4^ Within the m^6^A modification mechanism, our research highlights the indispensable role of the METTL3-CASP9/BIRC3 pathway in the progression of ESCC. Interestingly, we also found that the regulatory effects of METTL3 on *CASP9* and *BIRC3* are opposite in ESCC. We speculate that up-regulation of METTL3 may promote the advancement of ESCC through m^6^A modification of *CASP9* or *BIRC3* mRNA, but the modulation of their expression depends on downstream “m^6^A reader” proteins. Therefore, exploring the regulatory effect of “reader” proteins on m^6^A-methylated target mRNA will be a very meaningful study (Fig. S6). In conclusion, this pathway may represent a crucial regulatory axis that influences the behavior of ESCC cells, including their proliferation, survival, and response to PTX treatment. The pathway forms an intricate network of regulatory interactions and presents itself as a promising target for the diagnosis and therapeutic intervention of ESCC.

## CRediT authorship contribution statement

**Pengxiang Ji:** Writing – original draft, Validation, Investigation, Formal analysis, Data curation. **Bo Wan:** Writing – review & editing, Methodology, Investigation, Funding acquisition, Formal analysis, Conceptualization. **Minghui Gao:** Validation, Investigation, Data curation. **Shaohua Yin:** Validation, Software, Investigation. **Han Wu:** Validation, Formal analysis. **Junjie Wang:** Visualization, Software, Formal analysis. **Yuting Ma:** Funding acquisition, Data curation. **Weihua Xu:** Supervision, Project administration, Investigation, Funding acquisition, Conceptualization. **Minghua Wang:** Writing – review & editing, Supervision, Project administration, Methodology, Formal analysis, Data curation, Conceptualization.

## Ethics declaration

All aspects of this study were approved by the Institutional Research Ethics Committee of Soochow University (ECSU-201700033, ECSU-201700034) and conducted in accordance with the Declaration of Helsinki.

## Funding

This study was partially supported by the 10.13039/501100001809National Natural Science Foundation of China (No. 81872417, 81572923), the Priority Academic Program Development of Jiangsu Higher Education Institutions of China (PAPD), Social development Grant of Jiangsu Province, China (No. BE2023724), Youth Innovation of Promotion Association of Chinese Academy of Science (No. 2020323), and the Suzhou Municipal Science and Technology Bureau (Jiangsu, China) (No. SKYD2022074).

## Conflict of interests

The authors declared no conflict of interests.
