# Supplementary material for: METTL3 promotes esophageal squamous cell carcinoma progression and reduces chemosensitivity to paclitaxel through the CASP9/BIRC3-dependent apoptosis pathway
Source: Genes Dis. 2025 May 27;13(1):101693. doi: 10.1016/j.gendis.2025.101693 (PMC12624589; doi:10.1016/j.gendis.2025.101693)
Supplement: Multimedia component 1 [file mmc1.docx]

**Supplementary material for**

**METTL3 promotes esophageal squamous cell carcinoma progression and reduces chemosensitivity to paclitaxel though CASP9/BIRC3-dependent apoptosis pathway**

Ji Pengxiang^1,3^*, Wan Bo^1,2^*^#^, Gao Minghui^1^, Yin Shaohua^1^, Wu Han^1^，Wang Junjie^1^， Ma Yuting^4^, Xu Weihua^5,6#^, Wang Minghua^1,6#^

^#^**corresponding author:**

Wang Minghua ([mhwang@suda.edu.cn](mailto:mhwang@suda.edu.cn)),

Xu Weihua ([xuweihua2208@suda.edu.cn](mailto:xuweihua2208@suda.edu.cn)),

Wan Bo ([wanbo@suda.edu.cn](mailto:wanbo@suda.edu.cn)),

This file includes:

Supplementary Fig. S1 to S6

Supplementary Table. S1, S2, S3

Methods and Materials

Fig. S1


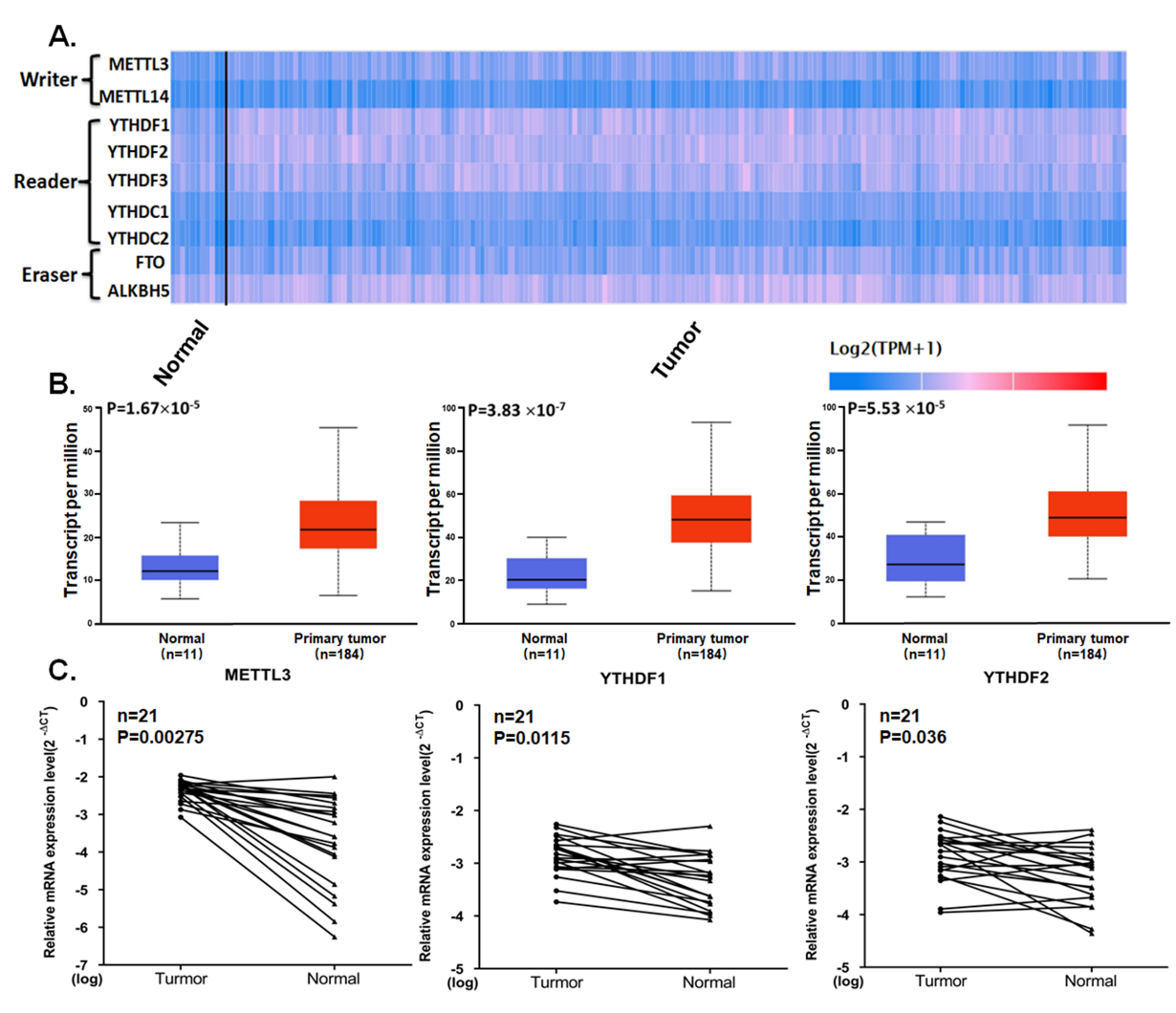


Fig. S1 Expression profiles (and clinical relevance) of RNA m^6^A regulators in ESCC.

(A) Heatmap of RNA m^6^A regulators expression from TCGA ESCC dataset, samples were grouped as normal and tumor tissues; (B) Relative METTL3, YTHDF1 and YTHDF2 mRNA expression in normal and tumor tissues from the TCGA ESCC dataset; (C) Validation of METTL3, YTHDF1 and YTHDF2 expression levels in adjacent tissues cancer and adjacent from 21 ESCC patients by qRT‒PCR. Data are presented as the mean ± S.D. Two-way ANOVA (B) and Student’s t-test (C) were used, **P* < 0.05, ***P*< 0.01, ****P* < 0.001.

Fig. S2


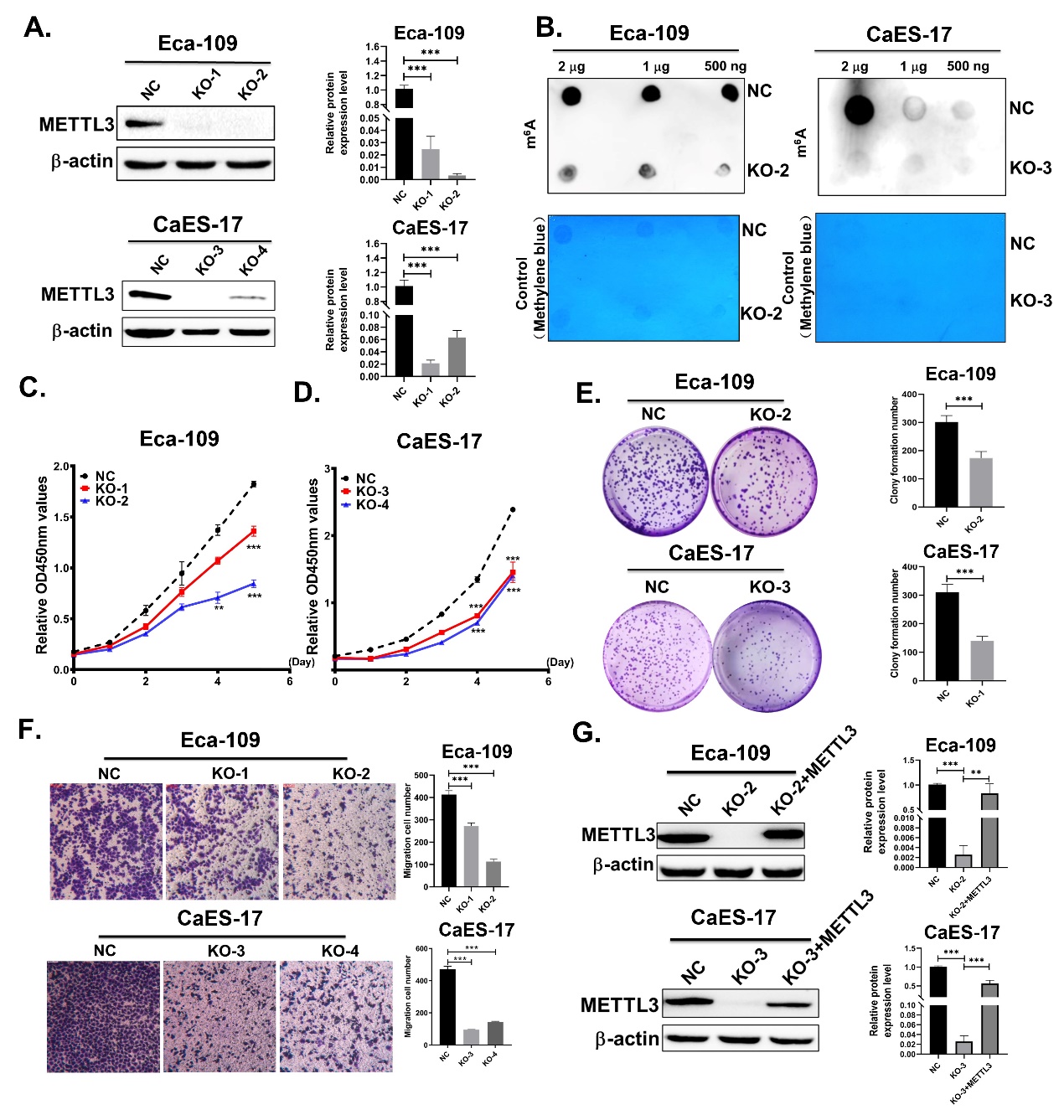
Fig. S2: Ablation of METTL3 inhibits proliferation and migration in ESCC cells.

(A) Western Blot was used to detect the knockout efficiency of METTL3 in Eca-109 and CaES-17 cells (n=3); (B) Validation of m^6^A-RNA methylation level in wide-type and METTL3-knockout of ESCC cell lines by Dot blot (n=3); (C-F) CCK8, crystal violet staining and Transwell assay were used to detect the proliferation and migration in wide-type and METTL3-knockout of ESCC. (n=4) (G) Western Blot was used to detect METTL3 expression in METTL3-KO Eca-109 and CaES-17 cells, METTL3-KO cells restoration with METTL3 expression (n=3). Data are presented as the mean ± S.D. Student’s t-test, **P* < 0.05, ***P*< 0.01, ****P* < 0.001.

Fig. S3


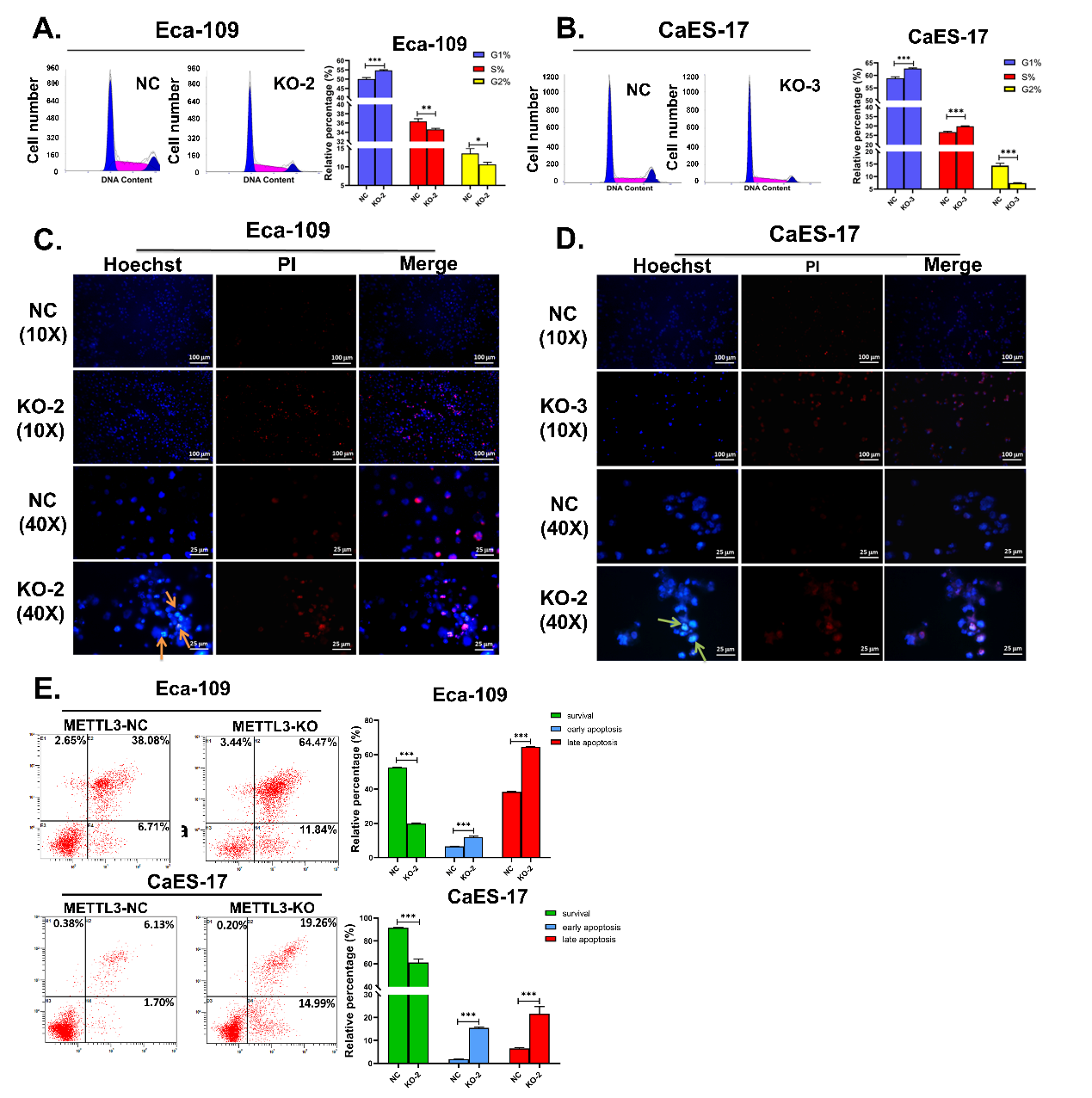


Fig. S3: Ablation of METTL3 promotes paclitaxel-induced cell cycle arrest and apoptosis in ESCC cells. (A, B) Flow cytometry was used to examine the effect of knockout of METTL3 on cell cycle in Eca-109 and CaES-17 cells; (C, D) PI/Hoechst double-staining assay of apoptosis levels in wide-type and METTL3-KO ESCC cells after PTX treatment (40 nM for Eca-109, 20nM for CaES-17). (E) Annexin V-FITC/PI apoptosis flowcytometry analysis was used to detect the apoptosis level in wide-type and METTL3-KO ESCC cells after PTX treatment (40 nM for Eca-109, 20nm for CaES-17). Data are presented as the mean ± S.D. n=3, Student’s t-test, **P* < 0.05, ***P*< 0.01, ****P* < 0.001.

Fig. S4


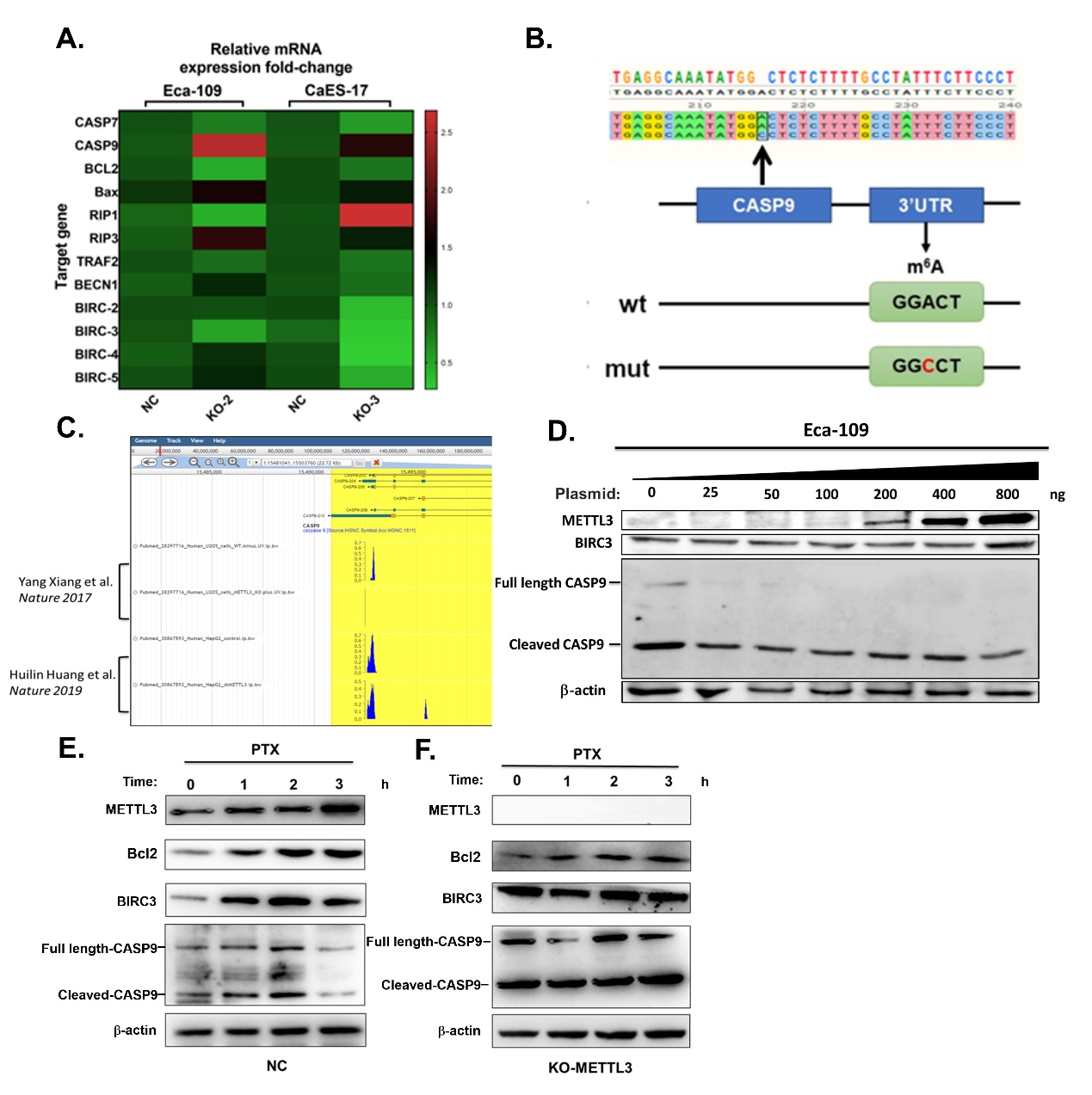


Fig. S4: METTL3 regulates CASP9/BIRC3 apoptosis-related genes expression in ESCC cells. (A) qPCR was used to detect the mRNA levels of apoptosis-related target genes in wide-type and METTL3-KO ESCC cells; (B, C) The m^6^A motif and peak distribution in CAPS9. （D）The influence of gradient transfection of METTL3 plasmid (0-800ng ) on the protein levels of METTL3, BIRC3, and CASP9.（E, F）The protein level of METTL3 and target genes were detected by western blot after the PTX treatment (40 nM) in NC and METTL3-KO group. Data are presented as the mean ± S.D. n=3, Student’s t-test, **P* < 0.05, ***P*< 0.01, ****P* < 0.001.

Fig. S5


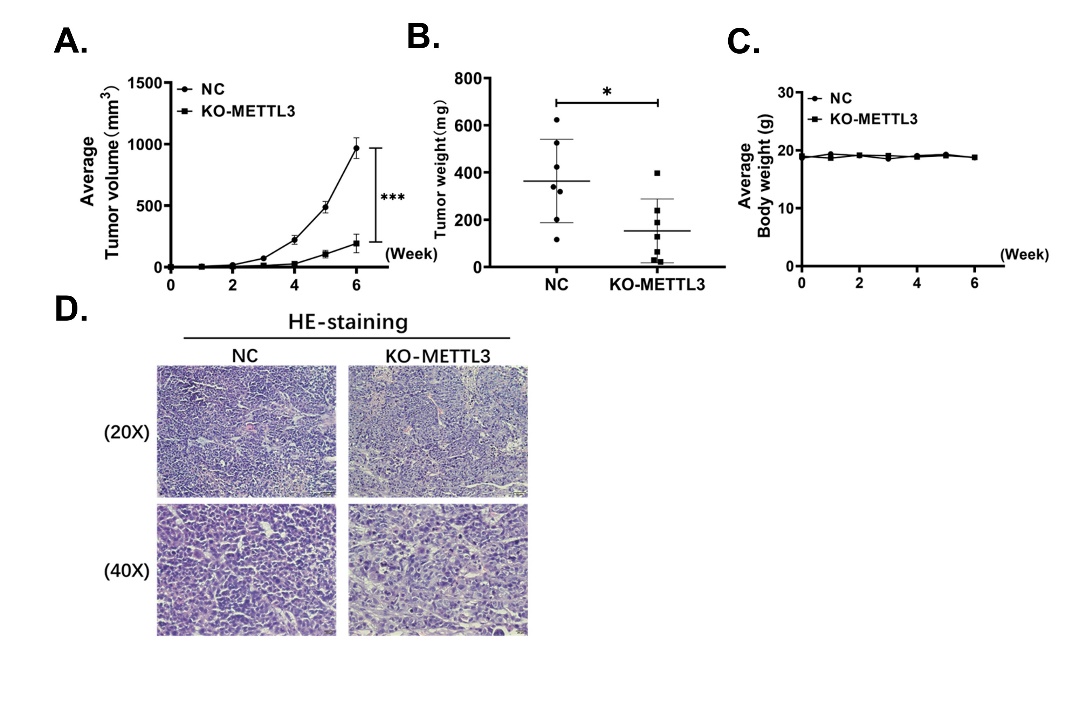


Fig. S5: METTL3 knockout inhibits Xenograft formation and inactivates apoptosis pathway in ESCC. (A) the tumor growth curve; (B) the weight of tumors xenografted in nude mice harvested 6 weeks after subcutaneous injection; (C) the weight growth curve; (D) H&E staining was used to detect cell morphology of xenografts tissues. Data are presented as the mean ± S.D. n=7, Student’s t-test, **P* < 0.05, ***P*< 0.01, ****P* < 0.001.

Fig. S6


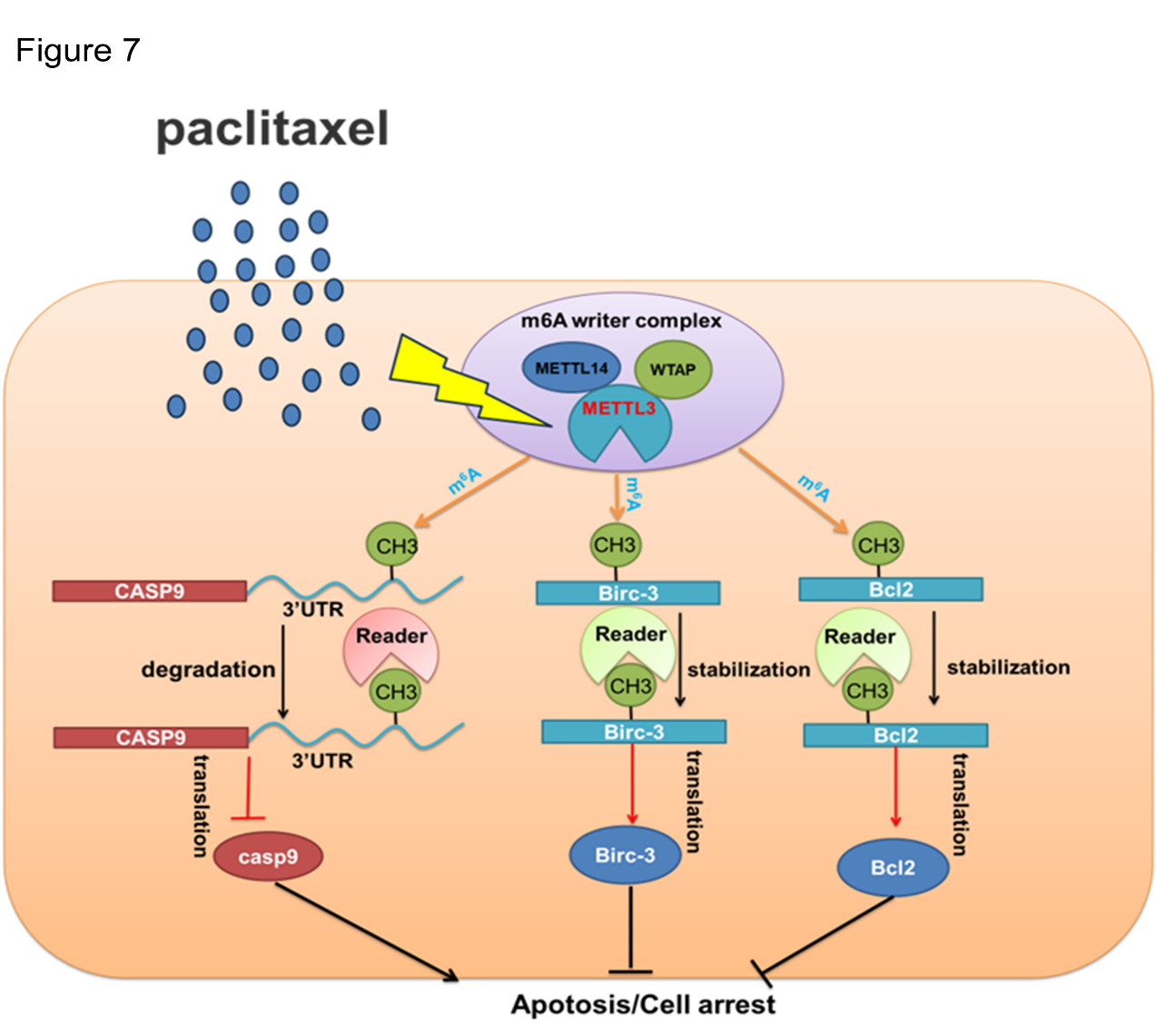


Fig. S6: The schematic diagram of the mechanism by which METTL3 in ESCC leads to paclitaxel resistance through inhibition of apoptosis.

Supplement Table S1. Sequences of guide RNA used to construct the CRISPR /Cas9 system.

| sgRNA | Sequence |
| --- | --- |
| sgMETTL3-1-F | CACCGGAGTTGATTGAGGTAAAGCG |
| sgMETTL3-1-R | AAACCGCTTTACCTCAATCAACTCC |
| sgMETTL3-2-F | CACCGGGGCTGTCACTACGGAAGGT |
| sgMETTL3-2-R | AAACACCTTCCGTAGTGACAGCCCC |

Supplement Table S2. The drug concentration and the corresponding cell viability test data are used to calculate the IC50.

| Eca-109 | | | | | | | | |
| --- | --- | --- | --- | --- | --- | --- | --- | --- |
| Concentration  (nM) | NC(Viability%) | | | | KO(Viability%) | | | |
| 0.00 | 107.03 | 103.51 | 96.08 | 93.38 | 101.40 | 101.35 | 100.18 | 97.07 |
| 20.00 | 82.38 | 76.71 | 78.64 | 77.92 | 61.32 | 62.65 | 62.88 | 60.65 |
| 40.00 | 69.12 | 65.87 | 64.22 | 60.97 | 50.65 | 52.54 | 52.20 | 49.26 |
| 80.00 | 60.09 | 57.67 | 57.23 | 53.65 | 43.25 | 44.14 | 45.09 | 43.86 |
| 160.00 | 52.99 | 47.98 | 47.05 | 46.77 | 39.03 | 38.14 | 38.53 | 36.08 |
| 320.00 | 44.79 | 42.87 | 43.91 | 41.55 | 34.41 | 33.80 | 33.36 | 33.86 |
| 640.00 | 43.42 | 41.33 | 40.72 | 40.34 | 31.97 | 32.02 | 31.63 | 30.63 |
| 1280.00 | 39.95 | 41.93 | 43.42 | 40.17 | 32.19 | 30.58 | 30.13 | 30.35 |

| CaES-17 | | | | | | | | |
| --- | --- | --- | --- | --- | --- | --- | --- | --- |
| concentration(nM) | NC(Viability%) | | | | KO(Viability%) | | | |
| 0 | 99.53 | 95.76 | 102.66 | 102.05 | 93.01 | 101.1 | 103.06 | 102.83 |
| 2.5 | 98.36 | 102.1 | 97.01 | 96.06 | 87.83 | 88.84 | 89.79 | 93.19 |
| 5 | 97.71 | 99.27 | 93.97 | 92.32 | 75.57 | 72.84 | 74.92 | 73.25 |
| 10 | 65.96 | 77.17 | 68.14 | 69.09 | 41.42 | 49.09 | 51.65 | 49.63 |
| 20 | 45.03 | 54.28 | 53.46 | 49.42 | 15.95 | 19.82 | 19.93 | 20.05 |
| 40 | 22.28 | 22.71 | 18.72 | 24.32 | 10.41 | 12.14 | 13.81 | 14.76 |

Supplement Table S3. Sequences of primers used for real-time PCR.

| Primer | Sequence |
| --- | --- |
| CASP7-F | CCTCGTTTGTACCGTCCCTC |
| CASP7-R | TTCCGTTTCGAACGCCCATA |
| CASP9-F | TAGTTTGCCCACACCCAGTG |
| CASP9-R | CGAAACAGCATTAGCGACCC |
| Bcl2-F  Bcl2-R | TGGGGTCATGTGTGTGGAGA  AGAAATCAAACAGAGGCCGCA |
| Bax-F | TCATGGGCTGGACATTGGAC |
| Bax-R | GCGTCCCAAAGTAGGAGAGG |
| RIPK1-F | CGGCCTTGCCTCCTTTAAGA |
| RIPK1-R | CCGACTTCTCTGTGGGCTTT |
| RIPK3-F | CTCAAGCCATCCAACGTCCT |
| RIPK-3-R | GAGGCCTTCCGGTTTACGTT |
| TRAF2-F | TGCGTATCTACCTGAACGGC |
| TRAF2-R | ACGTGCTCCCGGTTATTCTG |
| BIRC2-F | ACATGTGTGCTTCCTATCCTGG |
| BIRC2-R | GATGTTGGCCGCAGCATTTC |
| BIRC3-F | TGCAGCCCGCTTTAAAACAT |
| BIRC3-R | ACCTTGGAAACCACTTGGCA |
| BIRC4-F | ACTGAAGAGCAGCTAAGGCG |
| BIRC4-R | TGACTGTGTAGCACATGGGAC |
| BIRC5-F | CAAGGACCACCGCATCTCTA |
| BIRC5-R | ATGTTCCTCTATGGGGTCGTC |
| ACTB-F | GAAAATCTGGCACCACACCT |
| ACTB-R | ATAGCACAGCCTGGATAGCAA |

**Materials and Methods**

**Cell culture**

Esophageal squamous cell carcinoma cell lines Eca-109 and CaEs-17 cells (obtained from the Shanghai Institute for Biological Sciences) were cultured in RPMI 1640 medium containing 10% fetal bovine serum and 1% penicillin-streptomycin. HEK293T was cultured in DMEM high glucose medium in a cell incubator with 5% CO2 at 37 ° C.

**Cell transfection**

Adherent cells were collected and counted by a cell counting plate. A certain proportion of the cells was then inoculated into each well of a six-well plate, with a density of approximately 1×10^5-5×10^5 cells per well. Once the cells adhered to the plate, their density was observed under a microscope, and when it reached around 60%-70% density, transfection was performed. Before transfection, the cell culture medium was replaced with serum-free medium. The plasmid and PEI transfection reagent were separately mixed with serum-free medium in sterile centrifuge tubes, and then combined together and incubated for 20 minutes. The resulting mixture was then added to each well of the six-well plate and incubated for 4-6 hours. After transfection, the cells were observed under a microscope, and the serum-free medium was removed by suction with a negative pressure pump. Complete medium was then re-added to the cells.

**Establishment of Crispr/Cas9 knockdown system**

The CDS region of the target gene to be knocked out is selected according to the target gene, and the corresponding genomic DNA is searched on UCSC, and the corresponding exon fragments are found in gDNA, and each exon fragment is found. Logging on to https://www.benchling.com/crispr/ and https://www.genscript.com/grna-design-tool.htmL website design gRNA. The sequences of the oligos used in this study are shown in the Table S1. The oligos were then annealed following a standard protocol and ligated into the vector lentiCRISPR v2 (Addgene, #52961) and confirmed by sequencing. The plasmids were transfected into ESCC cell lines at 60% confluency for 36 h. Puromycin was added to the culture to select for transfected cells for 48h after transfection. The surviving cells were subjected to picking single clones by limiting dilution.

**Real-time fluorescent quantitative PCR**

The RNA was extracted by VeZol Reagent (vazyme), and after measuring the concentration and purity, 1μg of total RNA was reverse transcribed by HiScript III RT SuperMix for qPCR (vazyme). The transcribed RNA was then diluted according to proportion and used for subsequent quantitative PCR. The primers used in the PCR process were designed based on specific regions of the target gene according to NCBI. The expression levels of all genes were normalized to the reference gene, ACTB. The specific primer sequences are detailed in Table S3.

**m^6^A Dot Blot**

Total RNA was extracted and was denatured at 95 °C for 3min at various dilution concentrations to disrupt the secondary structure of the RNA. Immediately after denaturation, it is cooled on ice to prevent the secondary structure of RNA from re-forming; 2μL of the treated RNA was dropped directly onto the Hybond-N+ membrane used for nucleic acid blotting and allowed to dry. The membrane of the spot RNA was placed on a Stratalinker 2400 UV2400 violet tandem instrument for cross-linking (1200 microjoules [X100], 25-50 sec). Take a clean washing tray, add 10 mL of washing buffer to the tray, put the membrane after crosslinking into the tray, shaking slowly and wash for 5min at room temperature, put the membrane into the tray containing 10 mL of blocking solution, and incubate for 1 hour; After blocking, wash with TBST, shaking for 10-15min each time, a total of three times, remove TBST after washing; 10 ml of pre-prepared anti-M^6^A antibody dilution buffer (1:250 dilution; 2 μg/ml, SYSY, Germany), added to the plate containing the film, and shaken slightly overnight at 4°C; The incubated primary antibody was recovered, and the membrane was washed three times with 10 ml TBST buffer. 10 mL of pre-prepared goat anti-Rabbit IgG-HRP (1:10 000 dilution; 20 ng/ml) of secondary antibody dilution buffer was added to the plate containing the membrane and incubated for 1-2h at room temperature on a shaker；At the end of incubation, the secondary antibody was washed with TBST, shaking for 10-15min each time, a total of three times, and the TBST was removed at the end of washing. Hybond-n+ membranes were coated with 3 ml ECL luminescence solution at room temperature and incubated in the dark for 5 min. The film was wrapped in plastic packaging and properly exposed using a chemiluminescence meter.

**Cell proliferation assay/Drug addiction cell proliferation assay**

The stable strains were digested and prepared into cell suspension, and 1×10^3 cells were seeded in each well of 96-well plate. The experimental group and the control group were each set with 4 multiple Wells. In addition, PBS was added to the outermost circle of the 96-well plate to prevent the medium from evaporating. The 96-well plates inoculated with cells were cultured in a cell incubator with 5% CO2 concentration at 37 °C for 2-4h to make the cells adherent to the wall. After adherent, the 96-well plates were removed, and 10µL CCK8 was added to each well of the first group, and the cells were re-incubated in the cell incubator for 2h. After incubation at 37 °C for 2h, the 96-well plate was removed, and the absorbance value at 450nm wavelength was detected by microplate reader and used as the data on day 0. Then one of the 96-well plates was removed every 24 hours to repeat the CCK8 treatment and culture process, and the absorbance at 450nm wavelength was measured by microplate reader, which was used as the data of the first, second, third, fourth, and fifth day of cell culture. The data of 6 days were sorted out, and the cell growth curve was drawn with the cell culture time as the horizontal axis and the OD450 value as the vertical axis.

**Cell clone formation experiment**

The diluted cell counting plate was counted and diluted to a final concentration of 500 cells /mL according to the number of cells. The above cell suspension inoculation in 12 orifice, 1 ml per hole, the experimental group and control group each set three repeat, gently shaking, make the cells spread evenly, at 37 °C, 5% CO2 incubator let stand in the train, replace a piece every 3 ~ 4 days fresh medium, training 2 weeks left to old media, with the naked eye and microscope observation, After 2 weeks of culture, the old medium was discarded and observed by naked eye and microscope. If the cell clones grew to a suitable size, the cells were washed twice with PBS, and the PBS was discarded and 500μL of absolute methanol was added to cover the bottom of the culture plate, and the cells were fixed for 20 min. The fixation solution was removed, and 0.1% crystal violet staining solution was added for 20 min. Then the staining solution was washed off with slow water flow, and the cells were dried at room temperature. After drying, the images were taken and counted with ImageJ, and the results were analyzed by Graphpad.

**Cell migration assay**

The stable strains were digested by trypsin, counted by cell counting plate, and the cell concentration was adjusted to 5×10^5^/mL in 1mL. The chamber was placed in a 24-well plate with tweezers, and 600μL medium containing 20% serum was added to the lower chamber to avoid bubbles. 200 μL of evenly mixed cell suspension was added to the upper chamber. After 24h culture, the chamber was removed, and the old medium was absorbed and washed with PBS. The samples were fixed with methanol for 20min and stained with 0.1% crystal violet for 20 min. They were observed and photographed under a microscope and counted.

**Cell cycle assay**

Serum-free cells were treated for 24h to synchronize the cells, and then serum-containing medium was added for 48h. Digest the cells with trypsin, and gently blow to make the cells appear as a single state; The digested cells were blown and washed with pre-cooled PBS. The cells were quickly shaken with pre-cooled 70% ethanol and fixed overnight. Discard ethanol, add pre-cooled PBS and gently blow cells to disperse them; The samples were stained with PI at 37℃ for half an hour, and RNA enzyme was added to remove RNA. The cell cycle was detected by flow cytometry.

**Apoptosis assay**

Cells (approximately 5×10^5^ cells per test) were collected and then washed with cold PBS; The cells were suspended in 1 mL of 1×Binding Buffer, centrifuged in 300g for 10min, and then the binding buffer from cell precipitation was aspirated. The cells were resuspended in 1 mL Binding Buffer, and 100μL cell suspension (cell number controlled at 1×10^5^) was added to each labeled EP tube. 5μL Annexin V-FITC was added to the appropriate test tubes; Gently vortex each tube and incubate at room temperature for several minutes, out of light; Add 5μL PI solution and incubate for 5min in the room, away from light; Add 500μL PBS and swirl gently. The samples were detected and analyzed by flow cytometry within 1 hour.

**PI/Hoechst staining**

Inoculate 2×10^5^ cells into a six-well plate, discard the supernatant after adding drug treatment, add 10 μl Hoechst 33342 stock solution (Beyotime, China), and mix well; incubate at 37°C for 5-15 min; cool the cells on ice, add 5 μL PI stock solution (Beyotime, China), mix well, and re-stain at 37°C for 10-15 min; wash with PBS and observe with a fluorescence microscope.

**Western Blot**

Cells were lysed by lysis buffer (Beyotime) on ice for 30 min. The supernatant was collected after centrifugation at 1000g and the protein concentration in the lysate was detected by BCA assay kit (Beyotime). The loading buffer was added, and the mixture was heated for 10 min at 100°C. Proteins were resolved and transferred onto PVDF membranes. After blocked with 5% non-fat milk for 2 h at room temperature, the primary antibodies Were added and was shaken slowly overnight at 4°C. Primary antibodies targeting METTL3 (1:1000, Abcam, United Kingdom), CASP9 (1:1000, Proteintech, China), BIRC3 (1:1000, Proteintech, China), Bcl-2 (1:1000, Proteintech, China), and β-actin (1:10000, Proteintech, China) were employed. On day two, membranes were washed and incubated with secondary antibodies for 1 h. Protein signals were detected via an enhanced chemiluminescence system (Bio-Rad, United States).

**Xenograft tumor mouse model**

All animal experiments were approved by the Soochow University. Four-week-old male BALB/c nude mice (nu/nu; n = 7) (Soochow University Laboratory Animal Center, China) were anesthetized with an isoflurane/propylene glycol mixture, METTL3-KO Eca-109 cells and control cells were subcutaneously injected into the front of the right backside of mice (5.0 × 10^6^ cells in 100 μL PBS for one mouse). Mice weight, tumor length and width were measured per week for up to 6 weeks. The tumor sizes were assessed by measuring two dimensions, and the tumor volumes were calculated as the volume = (tumor length) × (tumor width) ^2/2. After 6 weeks tumor growth, tumor tissues were harvested for tumor weight measure, HE staining, and immunohistochemistry.

**Histology and immunohistochemistry**

Hematoxyline and Eosin (H&E) and Immunohistochemistry staining were performed as standard protocol. Briefly, Formalin-fixed tumor tissues harvested from xenograft tumor mouse model were embedded in paraffin, sectioned at 4 μm, and stained with hematoxylin and eosin (H&E). Primary antibodies against METTL3 (1:200, Abcam, United Kingdom), BIRC3 (1:400 dilution, Proteintech, China), CASP9 (1:400 dilution, Proteintech, China), KI-67(1:400 Abcam, United Kingdom) and HRP-conjugated secondary antibodies (MXB, Fuzhou, China) were used for immunohistochemical staining. The sections were counterstained with hematoxylin and photographed under a microscope.

**Statistical analysis**

All statistical analyses were performed using Prism 7.0 (GraphPad Software) and are presented as means ± SD. N numbers are reported in figure legends. The differences with different groups were determined by Two-way ANOVA or Student’s t-test, P< 0.05 was considered statistically significant.
